# A new cryptic species and review of the east-Andean leaf chafer genus *Mesomerodon* Ohaus, 1905 (Coleoptera, Scarabaeidae, Rutelinae)

**DOI:** 10.3897/zookeys.671.11815

**Published:** 2017-04-26

**Authors:** Matthias Seidel, Mary L. Jameson, Rachel L. Stone

**Affiliations:** 1 Department of Zoology, Faculty of Science, Charles University, Viničná 7, Praha 2, Czech Republic, CZ-12383; 2 Department of Entomology, National Museum, Cirkusová 1740, Praha 9, Czech Republic, CZ-19300; 3 Zentralmagazin Naturwissenschaftlicher Sammlungen, Martin-Luther-Universität Halle-Wittenberg, Domplatz 4, Halle (Saale), Germany, D-06108; 4 Department of Biological Sciences, Wichita State University 1845 Fairmount, Box 26, Wichita, KS, USA 67260-0026

**Keywords:** Amazonian lowland, pelidnotine chafer, sexual dimorphism, niche modeling, Selva baja amazónica, escarabajos pelidnotinos, dimorfismo sexual, modelado de nicho

## Abstract

The Neotropical scarab beetle genus *Mesomerodon* Ohaus (Scarabaeidae: Rutelinae: Rutelini) is distributed in the western (lowland) Amazonian region from Colombia to Bolivia. Based on our research, the genus includes three species including a new cryptic species from Ecuador. We use niche modeling to predict potential suitable habitat and identify environmental factors associated with the distribution of *Mesomerodon* species. We characterize the genus, provide a key to species, diagnose each species, describe a new species, provide spatial and temporal distributions, and discuss distributions of the species in relation to Amazonian landscape biodiversity.

## Abstract (es)

El género neotropical de escarabajos *Mesomerodon* Ohaus (Scarabaeidae: Rutelinae: Rutelini) se distribuye en las zonas bajas del oeste de la Amazonía, de Colombia a Bolivia. Con base en nuestro estudio, este género se compone por tres especies, incluyendo una nueva especie críptica de Ecuador. Utilizamos el modelado de nicho para predecir el hábitat adecuado potencial e identificar los factores ambientales asociados con la distribución de las especies de *Mesomerodon*. Aquí caracterizamos al género, proporcionamos una clave dicotómica para las especies, diagnosticamos cada especie, describimos una nueva especie, proveemos información sobre la distribución espacial y temporal de las especies y discutimos la distribución de las especies en relación a la biodiversidad de paisajes de la Amazonía.

## Introduction

**Figures 1–6.**
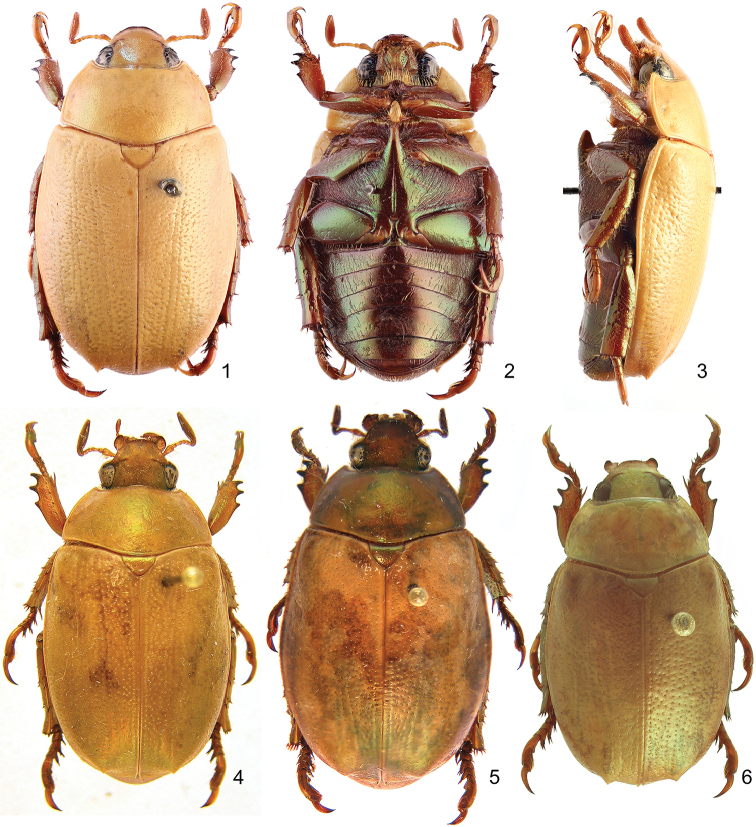
Dorsal and lateral habitus of *Mesomerodon* species. **1–3** *M. barclayi* sp. n., male holotype, dorsal, ventral, and lateral view **4–5** *M. gilletti* Soula holotype male specimen and female allotype specimen **6** *M. spinipenne* Ohaus male non-type specimen

**Figures 7–8.**
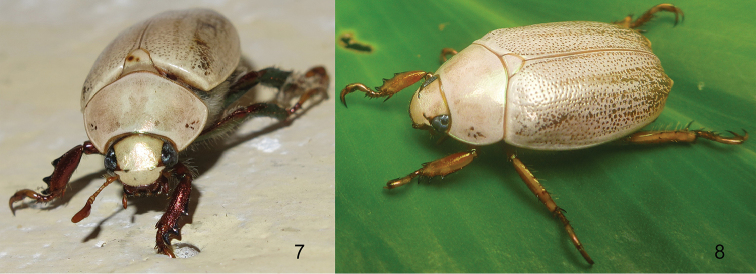
Live specimens showing cream coloration of *Mesomerodon* species. **7** *M. spinipenne* from Manú National Park, Madre de Dios, Peru [image courtesy of Rich C. Hoyer] **8** *M. barclayi* sp. n. from Payamino Research Station, Orellana, Ecuador [image courtesy of Conrad Gillett].

The South American genus *Mesomerodon* Ohaus (Figs 1–8) is a member of the pelidnotine leaf chafer scarabs, a polyphyletic assemblage of beetles which are in need of comprehensive revision (Moore et al. 2017). Members of the genus *Mesomerodon* are ovate, 17–25 mm in length, and cream-colored when alive (Figs 7–8). After death the color fades to testaceous or yellowish with weak metallic reflections. The genus is sexually dimorphic: males possess elongated, spinose elytral apices as well as an acute process on the posterior margin of the mesofemur (both lacking in females). The unusual form of the spinose elytral apex is a character state that is shared with males of the Neotropical leaf chafer *Hoplopelidnota metallica* (Laporte, 1840). Sister group relationships have not been addressed, host plant information is lacking, and larvae are undescribed. Members of this distinctive but poorly known group are distributed in lowland Amazonian regions (ca. 150–760 m elevation) from Colombia to Bolivia and are collected at light at night. In overall body form, the genus *Mesomerodon* resembles some species of *Pelidnota* MacLeay, 1819 (with which it is closely allied).

Ohaus (1905) established the genus *Mesomerodon* Ohaus and included in it one species with ‘peculiar sexual characteristics’ from Peru. Nearly 100 years after Ohaus’ (1905) description of the genus, Soula (2008) described a second species, *Mesomerodon gilletti* from Ecuador. In an overview of pelidnotine leaf chafers (Moore et al. 2017), the distribution of the genus was expanded to include Colombia and Bolivia. The distributional data provided for the genus *Mesomerodon* (Moore et al. 2017) were derived from the data in this study and are therefore given for the first time with specimen associations.

Species in the genus *Mesomerodon* possess many external similarities in form, but the male genitalia are sufficiently different as to warrant species status for three, distinct operational taxonomic units that we treat as species. Soula (2008) recognized *M. gilletti* as distinct from *M. spinipenne* based entirely on the form of the male genitalia (Fig. 23 versus Fig. 24). Cryptic species such as these are difficult or sometimes impossible to distinguish morphologically, and they are often incorrectly classified as a single taxon (Beheregaray and Caccone 2007, Bickford et al. 2007). Our synthesis of information on this group of beetles, which is based on 302 specimens and morphological data, led to the unveiling of an additional cryptic species in the genus.

We provide a synthesis of the biodiversity of the genus, including descriptions, key to species, diagnoses, and images. As a result of our research, the genus *Mesomerodon* includes three species, all of which are distributed in the western (lowand) Amazonia, including a new unexpected and cryptic species.

## Material and methods

**Characters.** Morphological characters formed the basis of this work. The broadest range of potentially phylogenetically informative morphological characters was used for morphological analyses and comparisons. Morphological terminology is based primarily on Jameson (1998), however we use the term venter instead of sternum and antennomeres instead of antennal segments. Antennomeres are defined as the pedicel plus flagellum (or flagellum and club). Consistent with use of venter, the term mesometasternum is replaced with mesometaventrum and the term sternite is replaced with ventrite. For measurements, we used an ocular micrometer. Body measurements, puncture density, puncture size, and density of setae are based on the following standards. Body length was measured from the apex of the clypeus to the apex of the pygidium. Body width was measured at the widest width of the elytra. Puncture density was considered ‘dense’ if punctures were nearly confluent to less than two puncture diameters apart, ‘moderately dense’ if punctures were from two to six puncture diameters apart, and ‘sparse’ if punctures were separated by more than six puncture diameters. Puncture size was defined as ‘small’ if punctures were 0.02 mm in diameter or smaller; ‘moderate’ if 0.02–0.07 mm, ‘moderately large’ if 0.07–0.12 mm, and ‘large’ if 0.12 mm or larger. Setae density was defined as ‘dense’ if the surface was not visible through the setae, ‘moderately dense’ if the surface was visible but with many setae, and ‘sparse’ if there were few setae. It should be noted that setae are subject to wear and may be abraded away. Elytral striae are defined as the striae located between the elytral suture and the elytral humerus. The interocular width measures the number of transverse eye diameters that span the width on the frons between the eyes. This was measured by placing the ocular micrometer in a position such that it intersects the frons and eyes (dorsal view), focusing on the surface of the frons, and then measuring the width of the frons and width of the eyes without adjusting the focus. Sclerotized portions of the male genitalia are used for diagnosis and identification. This includes the parameres, phallobase (or “basal piece” [d’Hotman and Scholtz 1990]), and the ventral sclerite of the phallobase (e.g., Fig. 22). Mouthparts, wings, and genitalia were examined and card-mounted beneath the specimen.

Characters and specimens were observed with 6–48× magnification and fiber-optic illumination. Digital images of specimens and structures were captured using the Leica Application Suite V3.8. Images were edited in Adobe Photoshop CS2 (background removed, contrast manipulated).

**Species concept.** Species are characterized by combinations of characters including the form the male protarsomeres and form of the male parameres in caudal and lateral views, and form of the ventral sclerite of the phallobase. Identification of female specimens required associated males from the same collecting event (place and date). We use the phylogenetic species concept (Wheeler and Platnick 2000) in this work: “A species is the smallest aggregation of (sexual) populations or (asexual) lineages diagnosable by a unique combination of character states.”

**Locality data.** Locality data for all specimens examined as part of this study were translated into decimal latitude and longitude using GoogleEarth (http://www.google.com/earth/index.html) and provided (Suppl. material 1). If latitude and longitude data were not included in label data or were too vague to be informative, then we searched for GPS data in GoogleEarth and GoogleMaps. Maps were generated by entering the coordinates into an Excel sheet. These files were subsequently used to construct distribution maps using an R script which plots the distribution points on an elevational map constructed using Global Land One-kilometer Base Elevation (GLOBE) data (Hastings et al. 1999). For species accounts, locality data are recorded for country, department and province (Bolivia), region and province (Peru), department (Colombia), or province (Ecuador). Additional locality data are provided (Suppl. material 1).

**Distribution modelling**. To model the potential distribution and to identify environmental factors associated with *Mesomerodon* species, we used the maximum entropy algorithm (MaxEnt; Phillips et al. 2006, Elith et al. 2011) for the species distribution modeling and followed the workflow of Fikáček et al. (2014). For occurrence data, we summarized all available records for all species of *Mesomerodon*. We ran three independent analyses: a genus-level distribution analysis that included data for all species and unidentified specimens, an analysis of the distribution of *Mesomerodon spinipenne*, and a combined analysis of the Ecuadorian and Colombian species (*M. gilletti* + *M. barclayi* sp. n.). A separate analysis for either *M. gilletti* or *M. barclayi* sp. n. was not conducted due to the few number of data points per species. Because two species occur in sympatry and in similar climatic conditions (*M. gilletti* and *M. barclayi* sp. n.), we concluded that the combined species analysis is justified. Furthermore, a combined analysis of the latter two species made it possible to include data points with unassociated species (female specimens which could not be identified). We employed the high-resolution climate data available in the Worldclim database (Hijmans et al. 2005) containing 19 layers of climatic variables. Analyses were performed in R (MaxEnt command in Dismo package). After mapping the ecological niche of the genus and/or species, prediction values were converted into binary values (presence and absence) using the threshold for maximum training sensitivity and specificity provided by the outputs of the resulting models (Figs 19–21).

**Type specimens.** Friedrich Ohaus provided a legacy for understanding the biodiversity of Rutelinae with over 170 published papers and research collections (for biography see Smith 2003). Perhaps due to concern with destruction of museums during World War II, Ohaus often labeled specimens as types long after publication (e.g., Kuijten 1988, 1992; Jameson 1990, 1998; Smith 2003). These erroneous type specimens can be recognized because label data are incongruous with data in the original description. As part of this research, we found 10 specimens that were labeled as type specimens. Six of these specimens do not belong to the syntype series of *M. spinipenne* (Suppl. material 1): one specimen from ZMHB, four specimens from ZSM, one specimen from NHMB). To reduce future confusion, these were labeled “NOT a type specimen of *Mesomerodon spinipenne*, Ohaus, 1905, des. Seidel 2016” (see “Remarks” for *M. spinipenne*”).

**Collections (Suppl. material 1).** This research is based on 302 specimens in 25 collections. The material examined in the present study is housed in the following collections and was provided by the curators and/or collections managers:

**AMNH** American Museum of Natural History, New York, USA (Lee Herman)

**BMNH** The Natural History Museum, London, United Kingdom (Max Barclay, Beulah Garner)

**CCECL** Musée des Confluences, Lyon, France (Cédric Audibert)

**CMNC** Canadian Museum of Nature Collection, Ottawa, Canada (Andrew Smith, François Génier)

**DBPC** Denis Bouchard Personal Collection, Autouillet, France

**DCCC** David Carlson Personal Collection, Fair Oaks, California, USA

**DJCC** Daniel Curoe Personal Collection, Palo Alto, California, USA

**FMNH** Field Museum of Natural History, Chicago, Illinois, USA (Alfred Newton, Crystal Maier)

**FSCA** Florida State Collection of Arthropods, Gainesville, Florida, USA (Paul Skelley)

**IRSNB** Institute Royal des Sciences Naturelles de Belgique, Brussels (Alain Drumont, Pol Limbourg)

**JWPC** Jim Wappes Personal Collection, San Antonio, Texas, USA

**LACM** Los Angeles County Museum of Natural History, Los Angeles, California, USA (Brian Brown, Weiping Xie)

**MLJC** Mary Liz Jameson Personal Collection, Wichita, Kansas, USA

**MNHN** Muséum National d’Histoire Naturelle, Paris, France (Olivier Montreuil)

**MSPC** Matthias Seidel Personal Collection, Prague, Czech Republic

**NHMB** Naturhistorisches Museum, Basel, Switzerland (Daniel H. Burckhardt)

**NMPC** Department of Entomology, National Museum (Natural History), Prague, Czech Republic (Jiří Hájek)

**QCAZ** Catholic Zoology Museum, Pontificia Universidad Catolica del Ecuador, Quito, Ecuador (Giovanni Onore)

**SLCC** Stephane LeTirant Collection, Montreal, Canada

**SMNS** Staatliches Museum für Naturkunde, Stuttgart, Germany (W. Schawaller)

**UASC** Museo de Historia Natural Noel Kempff Mercado, Santa Cruz de la Sierra, Bolivia (Julieta Ledezma Arias)

**UNSM** University of Nebraska State Museum, Lincoln, Nebraska, USA (Brett Ratcliffe, M. J. Paulsen)

**USNM** U.S. National Museum, Washington, D.C. (currently housed at the University of Nebraska State Museum for off-site enhancement) (Floyd Shockley and Dave Furth)

**ZMHB** Museum für Naturkunde, Leibniz-Institut für Evolutions-und Biodiversitätsforschung an der Humboldt-Universität, Berlin, Germany (Joachim Willers, Johannes Frisch)

**ZSM** Zoologische Staatssammlung des Bayerischen Staates, Munich, Germany (Michael Balke)

## Taxonomy

### Genus Mesomerodon

Taxon classificationAnimaliaORDOFAMILIA

Ohaus, 1905

Figs 1–6 , 7–8 , 9–17 , 18 , 19–21 , 22–24 , 25–27

Mesomerodon Ohaus, 1905: 319. Type species M. spinipenne Ohaus, 1905: 320–321 (by monotypy).

#### Description.

**Figures 9–17.**
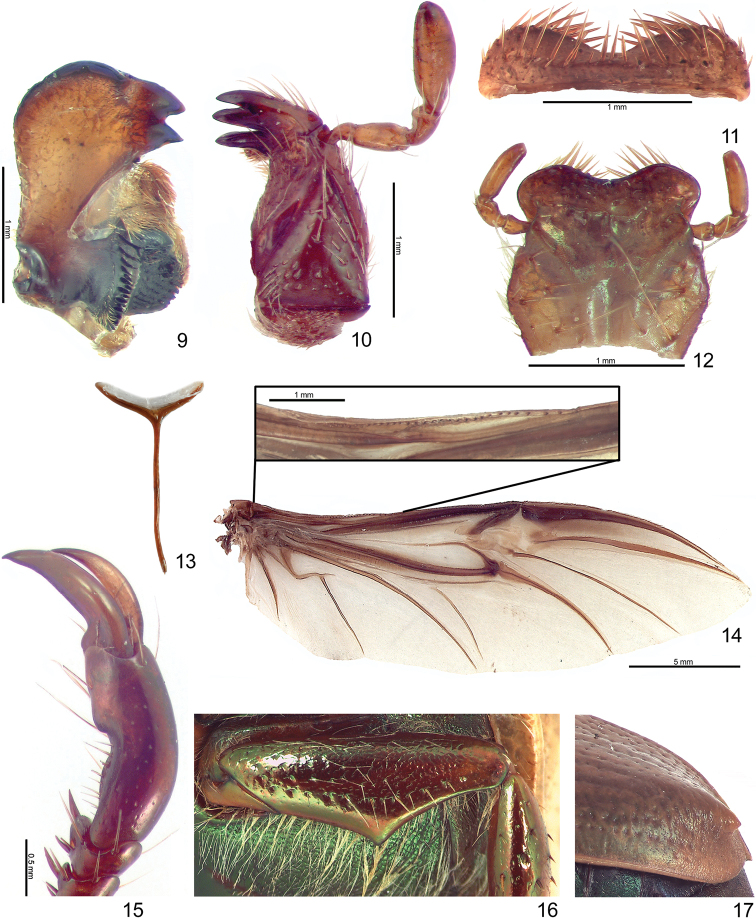
Generic characters for *Mesomerodon*. **9** Left mandible of *M. gilletti*, dorsal view (broadly rounded externally with 2 interior, acute teeth; molar region broad) **10** Maxilla of *M. gilletti*, ventral view (with 6 teeth; galea not fused) **11** Labrum, dorsal view, of *M. gilletti* (apex emarginate medially) **12** Mentum, ventral view, of *M. gilletti* (subrectangular in shape, broadest at middle, apex emarginated) **13** Spiculum gastrale of *M. gilletti* **14** Wing of *M. spinipenne* showing venation and inset showing dense, thick setae associated with ScA and setose region anterior to RA_3+4_ **15** Protarsomere 5 of *M. barclayi* sp. n., male, showing well defined, ventromedial emargination **16** Mesofemur of *M. gilletti* male, ventral view, showing acute process projecting posteriorly on posterior margin **17** Elytral apex of *M. gilletti*, lateral view, showing spiniform callus

Length from apex of clypeus to apex of pygidium 17.0–20.0 mm (♂) and 19.0–24.0 mm (♀); width at mid-elytra 10.0–12.0 mm (♂) and 11.0–14.0 mm (♀). Color: Dorsal surface tan to ochre (cream or whitish when alive) with or without weak green reflections, ventral surface castaneous with weak metallic green or red reflections; specimens tend to darken with age. Form (Figs 1–8): Elongate oval, widest at mid-elytra, pygidium exposed beyond apices of elytra; apices rounded with one short spine or tubercle near apex (males) or swelling (females). Head: Disc of frons and clypeus in lateral view flat, clypeus with margins and apex weakly reflexed; length of clypeus to frons (ratio) 0.5–0.6 : 1.0. Frons and clypeus moderately densely punctate, punctures small to moderate in size. Frontoclypeal suture weakly impressed, incomplete at middle. Eye canthus flattened, not weakly cariniform. Interocular width 2.5–3.8 transverse eye diameters. Clypeus rounded, with apex and lateral margin weakly reflexed, lacking bead; frontal view flat with short tawny setae, length (at middle) about 1/10 length of frons, disc moderately densely punctate, lacking setae. Mandible (Fig. 9) broadly rounded externally with 2 interior, acute teeth; molar region broad; mandibular apex always exposed. Labrum (Fig. 11) with apex emarginate medially, surface moderately densely punctate, punctures moderate in size, setose (setae moderately long and thick, tawny). Maxilla (Fig. 10) with 6 teeth; galea not fused, with moderately long setae. Mentum (Fig. 12) subrectangular in shape, broadest at middle, apex emarginated. Antenna with 10 antennomeres and 3-segmented club; club subequal in length to antennomeres 2–7 combined. Pronotum: Widest at base, apical angles acute, basal angles obtuse. Dorsal surface moderately densely punctate; punctures small and moderate in size. Bead complete anteriorly, laterally, and basally; setose basolaterally (setae moderately long, tawny or white). Scutellum: Parabolic, wider than long; base declivous at elytral base; dorsal surface as in pronotum. Wing (Fig. 14): Dense, thick setae present anterior to RA_3+4_ to apex; ScA with dense, thick setae near fold and with weak precostal pegs from near fold to base; AA_1+2_ shorter than AA_3+4_. Elytra: Surface punctate with weakly impressed striae; finely, densely rugose at apex. Punctures sparse to moderately dense, small to moderate in size, lacking setae. Sutural stria indicated by a row of punctures from base of scutellum to apex. Epipleuron from base to metacoxa with shelf and associated setae; beaded. Apex of elytra (Fig. 17) weakly rounded; elytral callus with well-defined tubercle or spine; sutural apex spiniform. Elytral sutural length about 10 times length of scutellum. Propygidium: Hidden beneath elytra. Pygidium: Subtriangular, about twice as wide as long at middle; finely, densely rugopunctate. Margins beaded with sparse, moderately long setae; setae tawny. Apex rounded. Venter (Figs 2–3): Prosternal process elongate-oval, projecting anteroventrally at about 35° with respect to ventral plane; apex produced to level of protrochanter, rounded; surface posteriorly protuberant in basal 1/4, with setaceous punctures; setae long, dense, tawny. Mesometaventral process produced anteriorly to prosternal process; apex acuminate, rounded; ventral surface weakly recurved toward apex in lateral view, lacking setae apically, with moderately dense setae basally (setae moderately dense, moderately long, tawny). Abdominal ventrites 1–4 subequal in length in male and female, ventrite 5 about 1.5–2 times the length of ventrite 4, ventrite 6 subequal in length to ventrite 4 (male) or 1.3 times length of ventrite 4 (female). Abdominal ventrites 1–4 subequal in length in male and female, ventrite 5 about 1.5–2 times the length of ventrite 4, ventrite 6 subequal in length to ventrite 4 (male) or 1.3 times length of ventrite 4 (female). Last ventrite with widest width to median length in males about 5.5:1 and in females about 4.3:1; surface smooth (male) or rugose (female). Last ventrite (male) subequal in length to ventrite 5, quadrate at subapex, subapical corners not produced, surface moderately densely punctate; region from subapex to apex less sclerotized, surface smooth. Last ventrite (female) subequal to ventrite 4, apex trapezoidal, surface rugose. In lateral view, male ventrites flat, female ventrites weakly convex. Legs: Protibia with 3 external teeth subequally separated in apical half; spur present, subapical; inner base lacking protibial notch. Protarsomere 5 of male a little longer than tarsomeres 1–4 and with well-defined ventromedial emargination (Fig. 15). Modified foreclaw of male 1.5–2 times width of unmodified claw, inner subapical tooth present, small. Foreclaws of female simple, internal claw as wide as outer claw. Unguitractor plate laterally flattened, weakly exposed beyond tarsomere 5; apex with 2 short setae. Protarsomere 2 (male) with or without striated region at ventral apex; lacking in female. Mesofemur with acute process projecting posteriorly on posterior margin (male) (Fig. 16). Mesotibia with sides subparallel, apex weakly divergent or parallel; external edge with 2 weak carinae (female), less pronounced in male; inner apex with 2 spurs; apex with 12–15 spinulae. Meso- and metatarsomere 4 apicomedially with 1 outer spinose seta and 1 inner stout spinose seta (male and female). Meso- and metatarsomere 5 with weak, triangular interomedial tooth or swelling. Outer claw of meso- and metatarsal claws slightly longer than inner claw; outer mesotarsal claw twice as wide as inner claw in males, subequal in width in females; metatarsal claws subequal in width; claws simple. Metatibia with sides subparallel, weakly divergent towards apex; external edge with 1–2 weak carinae (slightly more robust in female); inner apex with 2 spurs; inner apex with 14–26 short, stout spinulae. Metacoxal corner (female) weakly produced or square. Spiculum gastrale (Fig. 13): Weakly Y-shaped (arms ~30 degree angle), lacking associated sclerites and setae. Male genitalia (Figs 22–24): Parameres less than twice length of phallobase. Parameres fused dorsoventrally (not laterally), asymmetrical; diagnostic, species specific (Figs 22a, 23a, 24a). Ventral sclerite of phallobase asymmetrical or symmetrical, elongate (produced to apex of phallobase), apex subquadrate; diagnostic, species specific (Figs 22c, 23c, 24c). Female genitalia: Gonocoxites subtriangular to subquadrate with sparse setae; not diagnostic for species.

#### Natural history.

Biology for species in the genus is not known. Adults likely feed on plant foliage, but no host has been recorded. Because adults are attracted to lights at night, it is likely that feeding occurs at night. Larvae are not described, but likely feed on compost and/or roots.

#### Etymology.

From the Greek, “*mesos*” meaning middle or in the middle, “*mero*” meaning femur, and “*odon*” meaning tooth. The name refers to the spinose process on the posterior margin of the mesofemur in males, a synapomorphy for species in the genus. The gender is neuter.

#### Composition and distribution

**Figure 18.**
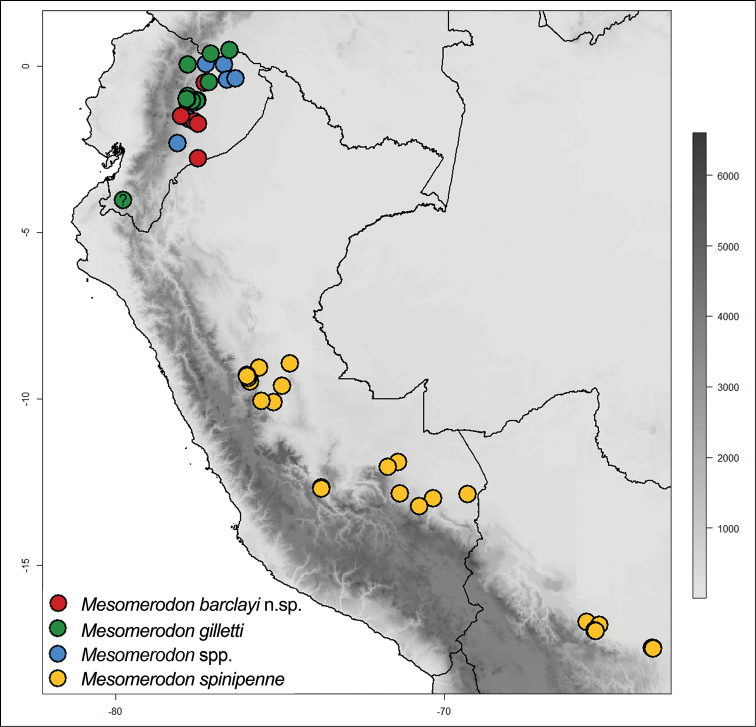
Distribution of *Mesomerodon* species in South America. Refer to Suppl. material 1 for associated data.

(Fig. 18). Three species distributed on western (lowland) Amazonia from Colombia, Ecuador, Peru, and Bolivia. An erroneous record from Brazil (Ohaus 1905) was repeatedly cited by subsequent authors (Blackwelder 1944, Ohaus 1934, 1952, Machatschke 1972, Krajcik 2008, Soula 2008, Moore et al. 2017) (see *Mesomerodon spinipenne* type material). We record the genus from elevations between 150 to 762 m. A record of the genus occurring at 2800 m (Paucar-Cabrera 2005) is beyond the limits of the genus, and we consider it erroneous. A locality record of *M. gilletti* from Loja (Ecuador) (Fig. 18 [indicated with question mark]) waits for confirmation through future collecting since a short series of specimens supposedly collected from Loja province (west side of the Andes) seems to be out of the altitudinal and longitudinal reach of the genus. The ranges of two species of *Mesomerodon* overlap in aseasonal Ecuador in a region known for the highest levels of mammal and plant species diversity (Hoorn et al. 2010). Rutelinae biodiversity in Ecuador is the highest recorded in South America, with 53 genera and 298 species (Paucar-Cabrera 2005). Of these, 92 species of Rutelinae (or 36%) are endemic to the country (Paucar-Cabrera 2005). The distribution of the genus is restricted to low elevations alongside the Andes without extending eastward into the Brazilian Amazon. *Mesomerodon* exhibits a distributional gap between *M. spinipenne* and the Ecuadorian species in northern Peru.

#### Niche modeling.

**Figure 19–21.**
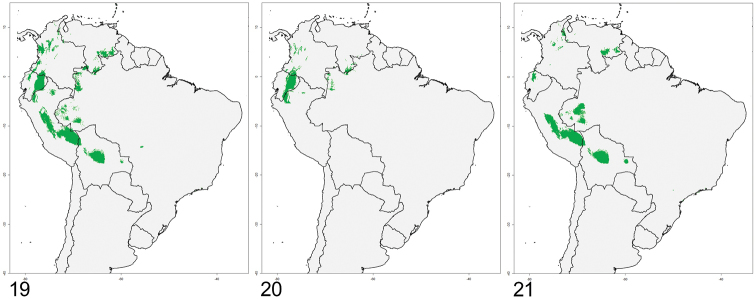
Distributional model for *Mesomerodon* species in South America. **19** all *Mesomerodon* species **20** *M. barclayi* sp. n., *M. gilletti* and unassociated females **21** *M. spinipenne*. Refer to Appendix 1 for associated data

Within the Andean corridor, the genus level distribution model is congruent with the specimen-based distribution (Fig. 19 vs. Fig. 18). Therefore, the apparent distributional gap between *Mesomerodon spinipenne* and the Ecuadorian species in northern Peru is unlikely a result of a lack of sampling. The distribution model of the Ecuadorian species (Fig. 20) suggests that either *M. barclayi* sp. n. or *M. gilletti* extend into northern Peru. The distribution model for *M. spinipenne* (Fig. 21) shows a continuous distribution in central and southern Peru with a disconnected population in Bolivia (also corroborated with the specimen-based distribution). Specimen-level data do not corroborate the occurrence of *M. spinipenne* in western Brazil, northern Colombia, or Venezuela. Collecting may reveal occurrence of the taxon in western Brazil, but we consider it unlikely that the taxon occurs in Colombia or Venezuela because these countries have been well collected.

#### Diagnosis.

**Figures 22–24.**
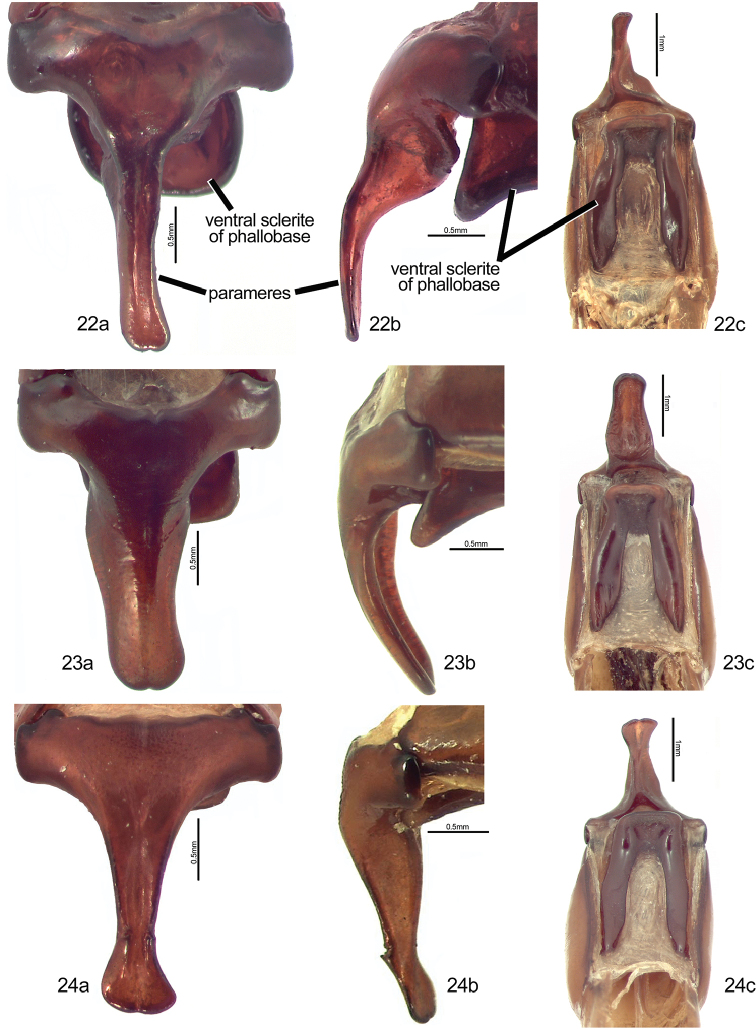
Form of the male genitalia (dorsal [**a**], lateral [**b**], ventral [**c**] views) in *Mesomerodon* species. **22** *M. barclayi* sp. n. **23** *M. gilletti* **24** *M. spinipenne*

Species in the genus *Mesomerodon* are distinguished from other pelidnotine leaf chafers based on the acute, spiniform processes on the apical callus of the elytra in males (Fig. 17; shared with *Hoplopelidnota*) and an acute process on the posterior margin of the mesofemur in males (Fig. 16; autapomorphic for the genus). The ovate body form, size, and color are similar to some species of Pelidnota (Pelidnota) (e.g., *Pelidnota lucida* Burmeister, 1844), but the form of the mandible clearly separates the two genera (*Mesomerodon* species possess a rounded mandibular apex [Fig. 9] whereas *Pelidnota* species possess a bidentate, reflexed mandibular apex). Additional characters that assist with diagnosis of *Mesomerodon* include: external edge of protibia with three teeth; pronotum with bead complete anteriorly, laterally and basally; mesoventrite produced beyond mesometaventral suture (Fig. 3); and male genitalia with highly sclerotized ventral sclerite of the phallobase (Figs 22c, 23c, 24c). See Moore et al. (2017) for a key to genera of pelidnotine scarabs.

**Key to *Mesomerodon* species**

**Table:** 

1	Mesofemur without process projecting posteriorly on posterior margin (♀)	**2**
–	Mesofemur with acute process projecting posteriorly on posterior margin (Fig. 16) (♂)	**3**
2	Distributed in Bolivia and Peru (Fig. 18)	***M. spinipenne* (♀) Ohaus**
–	Distributed in Ecuador and Colombia (Fig. 18)	***M. gilletti* (♀) Soula or *M. barclayi* (♀) Seidel, Jameson, & Stone, sp. n.**
3	Protarsomere 2 ventrally with striate region at apex (Fig. 27)	***M. spinipenne* (♂) Ohaus**
–	Protarsomere 2 ventrally lacking striate region at apex (Figs 25–26)	**4**
4	In lateral view, parameres curved posteriorly (Fig. 23b)	***M. gilletti* (♂) Soula**
–	In lateral view, parameres sinuate (Fig. 22b)	***M. barclayi* (♂) Seidel, Jameson, & Stone, sp. n.**

**Clave para las especies de *Mesomerodon***

**Table:** 

1	Mesofémur sin proceso proyectándose posteriormente en el margen posterior	**2**
–	Mesofémur con un proceso agudo proyectado posteriormente en el margen posterior (Fig. 16)	**3**
2	Distribuido en Bolivia y Perú (Fig. 18)	***M. spinipenne* (♀) Ohaus**
–	Distribuido en Ecuador y Colombia (Fig. 18)	***M. gilletti* (♀) Soula o *M. barclayi* (♀) Seidel, Jameson, & Stone, sp. n.**
3	Protarsómero 2 ventralmente con una zona estriada en el ápice (Fig. 27)	***M. spinipenne* (♂) Ohaus**
–	Protarsómero 2 ventralmente sin una zona estriada en el ápice (Fig. 25–26)	**4**
4	En vista lateral, parámeros curvados posteriormente (Fig. 23b)	***M. gilletti* (♂) Soula**
–	En vista lateral, parámeros sinuados (Fig. 22b)	***M. barclayi* (♂) Seidel, Jameson, & Stone, sp. n.**

### Mesomerodon barclayi

Taxon classificationAnimaliaORDOFAMILIA

Seidel, Jameson, & Stone sp. n.

http://zoobank.org/4B70B243-1A6A-445D-9C33-5E859DE8B086

Figs 1–3 , 8 , 15 , 18 , 22 , 25

#### Type material.

Holotype male and 19 paratypes (10 males, 9 females). Holotype male at ZMHB with label data: a) “Rutelide, Pacayacu, 23.8.37” (handwritten), b) “Mesomerodon spinipenne Ohs., MNHUB Berlin” (typeset), c) male genitalia card mounted, d) our red holotype label. Paratype female at MSPC with label data: a) “Ecuador, Pacayacu, 3.X.37, Dr. Schultze – Rhonhof S.G.” (typeset and handwritten), b) “Pacayacu 3.10.37” (handwritten), c) “loan from Zool.Mus. Berlin” (typeset), d) “Mesomerodon spinipenne Ohs., MNHUB Berlin” (typeset), e) our yellow paratype label. Paratype male at ZMHB with label data: a) ”O.ECUADOR, Sarayacu Feyer” (typeset), b) “Mesomerodon spinipenne, Cotype ♂ Ohs.” (handwritten on red label), c) “kein typus” (handwritten), d) male genitalia card mounted e) our yellow paratype label. Paratype female at ZMHB with label data: a) “O.ECUADOR, Sarayacu Feyer” (typeset), b) “Mesomerodon spinipenne, Cotype ♀ Ohs.” (handwritten on red label), c) “kein Typus” (handwritten), d) our yellow paratype label. Paratype male at ZMHB with label data: a) “O.ECUADOR, Sarayacu Feyer” (typeset), b) “88882” (typeset), c) “♂” (typeset on black bordered label), d) “Mesomerodon spinipenne Ohs., MNHUB Berlin” (typeset), e) male genitalia card mounted, f) our yellow paratype label. Paratype female at ZMHB with label data: a) “Rutelide u. Cyclocephala, Pacayacu 24.9.37” (handwritten), b) “Mesomerodon spinipenne Ohs., MNHUB Berlin” (typeset), c) our yellow paratype label. Paratype female at ZMHB with label data: a) “O. Ecuador, Puyo, A. Schulze” (typeset), b) “Ohaus determ., Mesomerodon spinipenne ♀ Ohs.” (typeset and handwritten), c) our yellow paratype label. Paratype female at ZMHB with label data: a) “O. Ecuador, Puyo, A. Schulze” (typeset), b) “Mesomerodon spinipenne Ohs.” (handwritten), c) “♀” (typeset), d) our yellow paratype label. Paratype male at CCECL with label data: a) ECUADOR (Orellana), Payamino Research Station, 0°29'967"S, 77°17'083"W, 300m Tropical Rainforest, At M.V. light at night, 30.vii–12 viii.2007” (typeset), b) male genitalia card mounted, c) our yellow paratype label. Paratype male at MSPC with label data: a) ECUADOR (Orellana), Payamino Research Station, 0°29'967"S, 77°17'083"W, 300m Tropical Rainforest, At M.V. light at night, 30.vii -12 viii.2007” (typeset), b) “coll. CPDT Gillett, BMNH(E) 2007-65” (typeset), c) “DNA extract No: Meso1, deposited at MSPC” (handwritten), d) male genitalia card mounted, e) “Matthias Seidel Collection 2016”, f) our yellow paratype label. Paratypes (2 males) at BMNH with label data: a) ECUADOR (Orellana), Payamino Research Station, 0°29'967"S, 77°17'083"W, 300m Tropical Rainforest, At M.V. light at night, 30.vii -12 viii.2007” (typeset), b) “coll. CPDT Gillett, BMNH(E) 2007-65” (typeset), c) “DNA extract No: Meso2, deposited at MSPC; no PCR amplification” (handwritten), d) male genitalia card mounted, e) our yellow paratype label. Paratype male at SLCC with label data: a) ”PARAGUAY: Alto Parana, 1.XI.1990” (typeset), b) “Collection S. Le Tirant” (typeset), c) “supposedly mislabeled locality, des. Seidel 2016” (typeset), d) “MESOMERODON SPINIPENNE OHAUS, det. M.E. Jameson 2003” (typeset and handwritten), d) male genitalia card mounted, e) our yellow paratype label. Paratype male at IRSNB with label data: a) ”ECUADOR (Orellana), Payamino Territory, 300m Tropical Rainforest, July 2007” (typeset), b) “DNA extract: Rut105, det. M.Seidel 2016” (typeset and handwritten), c) male genitalia card mounted, d) our yellow paratype label. Paratype male at ZSM with label data: a) ”O.Ecuador, Puyo, A. Schulze, 6.V.35” (typeset and handwritten), b) “Mesomerodon spinipenne Ohs.” (handwritten), c) “Staatssammlung München, 1975, Erwerb Coll. Machatschke”, d) male genitalia card mounted, e) our yellow paratype label. Paratype female at NMPC with label data: a) ”O.Ecuador, Puyo, A. Schulze, 6.V.35” (typeset and handwritten), b) “Ohaus determ., Mesomerodon spinipenne ♀ Ohs.” (typeset and handwritten), c) “Staatssammlung München, 1975, Erwerb Coll. Machatschke”, d) our yellow paratype label. Paratype female at ZSM with label data: a) ”O.Ecuador, Sarayacu Feyer” (typeset), b) “Paratypus, Mesomerodon spinipenne Cotype ♀ Ohs.” (typeset and handwritten), c) “Staatssammlung München, 1975, Erwerb Coll. Machatschke”, d) “NOT a type specimen of *Mesomerodon spinipenne*, Ohaus, 1905, des. Seidel 2016” (typeset), e) our yellow paratype label. Paratype male at FMNH with label data: a) ”ECUADOR: Pastaza; 300m confluence R. Macuma & R. Morona VII:17:1971, leg. B. Malkin” (typeset), b) “at light” (typeset), c) “on sand river bank” (typeset), d) male genitalia card mounted, f) our yellow paratype label. Paratype female at FMNH with label data: a) ”ECUADOR: Pastaza; 300m confluence R. Macuma & R. Morona VII:17:1971, leg. B. Malkin” (typeset), b) “at light” (typeset), c) “on sand river bank” (typeset), d) “Mesomerodon spp??? DET. A.R.Hardy 1980” (typeset and handwritten), f) our yellow paratype label. Paratype female at FMNH with label data: a) ”ECUADOR: Pastaza; 300m confluence R. Macuma & R. Morona VII:17:1971, leg. B. Malkin” (typeset), b) our yellow paratype label.

#### Description

**Figures 25–27.**
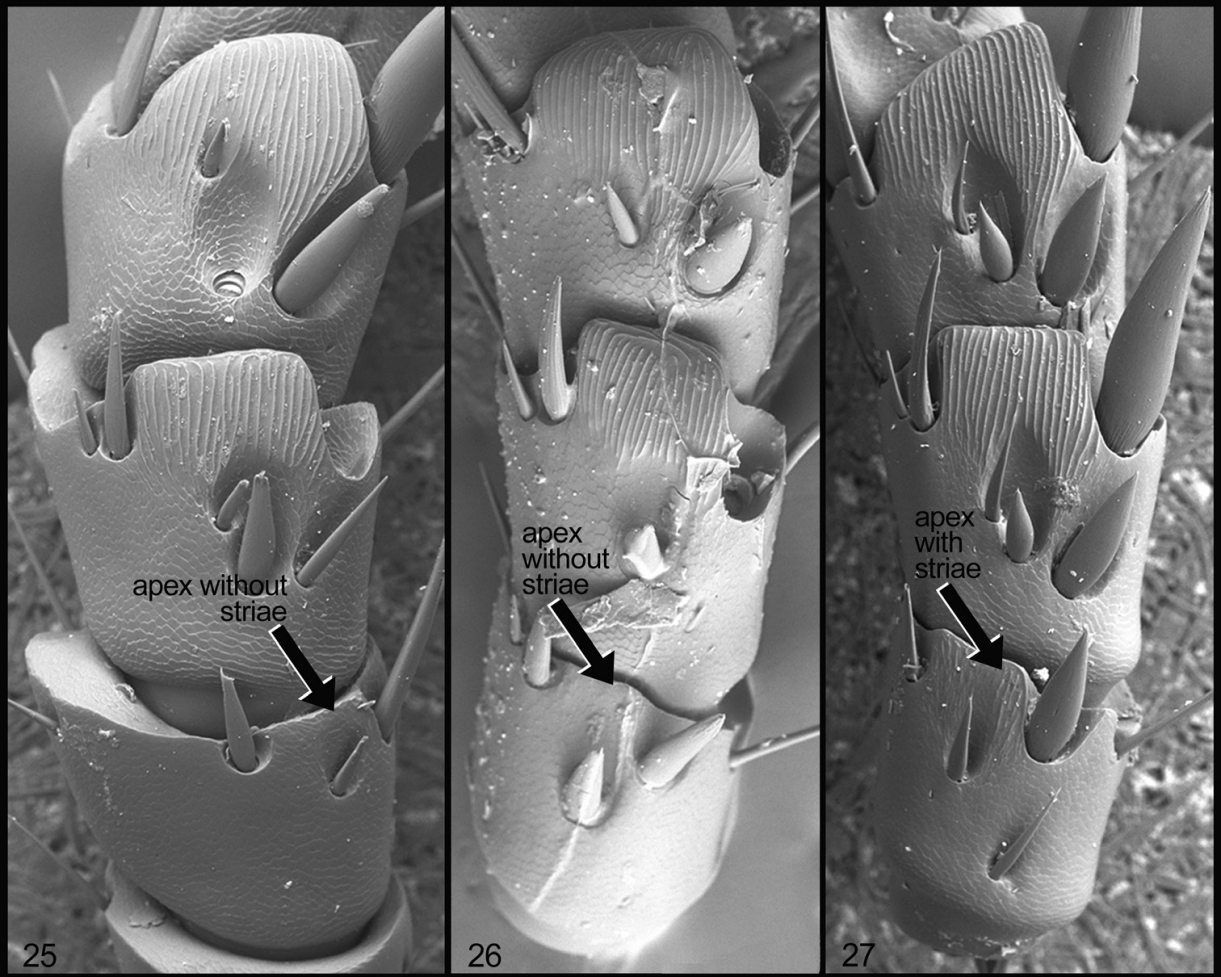
Form of protarsomeres 2 to 4, ventral view, in *Mesomerodon* species. **25** *M. barclayi* sp. n., showing protarsomere 2 without stiate region at apex **26** *M. gilletti* showing protarsomere 2 without striate region at apex **27** *M. spinipenne* showing protarsomere 2 with striate region apically.

**(based on 11 males and 9 females).** The holotype does not differ significantly from the generic description and suffices for the description of this cryptic species (see “Remarks”). Descriptive details specific to the holotype specimen are indicated. Length from apex of clypeus to apex of pygidium 18–22 mm (♂) (holotype: 19 mm) and 22–25 mm (♀); width at mid-elytra 11–12 mm (♂) (holotype: 11 mm) and 12–15 mm (♀). Color: Cream colored (holotype), tan, or ochre; ventral surface castaneous with weak metallic green reflections. Form: Elytral apices with one short spine (holotype) or swelling (females). Legs: Protarsomere 2 of male ventrally lacking well-defined striae at ventral apex (Fig. 25). Male genitalia (Fig. 22a–c): Parameres with elongate, narrow projection (=stem) and with longitudinal, impressed fissure (Fig. 22a); stem gradually and weakly broadened toward apex, subapex lacking paired, spinose projections; ventral sclerite of phallobase with surface concave, apex quadrate (Fig. 22c); lateral view diagnostic (Fig. 22b).

#### Diagnosis.

Males of *Mesomerodon barclayi* sp. n. are differentiated from other *Mesomerodon* species by the following combination of characters: Protarsomere 2 ventrally lacking a striated region at the ventral apex (striated in *M. spinipenne*; apical region lacking striae in *M. gilletti* [Figs 25–27]) and form of the male genitalia (Fig. 22 versus Fig. 24 in *M. spinipenne* and Fig. 23 in *M. gilletti*). Females can only be confidently determined when associated to male specimens from the same collecting event.

#### Etymology.

It is our honor to dedicate this species to Max Barclay (Curator, Coleoptera, Department of Entomology), who invited the first and second authors to the 1^st^ Scarab Symposium at the BMNH in 2014 where cooperation on this work was initiated. Max Barclay has led the way in making biodiversity science more accessible to scientists and citizens alike.

#### Distribution

(Fig. 18). Known from western (lowland) Amazonia areas in Ecuador and occurring in apparent sympatry with *M. gilletti*. Ohaus (1934, 1952) recorded *M. spinipenne* from Sarayacu, Ecuador. We recovered these specimens in ZMHB and ZSM and identified them as belonging to *M. barclayi* sp. n.

#### Locality data

**(Suppl. material 1).** 20 specimens from 9 collections.

ECUADOR: Morona-Santiago, Orellana, Pastaza

#### Temporal data.

Based on label data, this species is known to be active in the months of February, August, and November.

#### Natural history.

Based on label data, adult *M. barclayi* sp. n. is usually collected at light, thus suggesting activity and feeding at night. Individuals probably occur throughout the year. Immature stages are unknown. Specimens have been recorded at an elevation of 300 m.

#### Remarks.

As with other cryptic species, the overall body form of *M. barclayi* sp. n. is similar to other species in the genus. The form of the male genitalia (Fig. 22 versus Figs 23–24) however, is sufficient for identification of *M. barclayi* sp. n. When developing our species hypotheses, we examined this morphotype in terms of phenotypic, elevational, and seasonal variation within *M. spinipenne* or *M. gilletti*, but the consistency in the form of the male genitalia led us to conclude that it is a justified species. Apparent sympatry (in location and phenology) with *M. gilletti* also led us to carefully examine this species pair. Again, we found that consistency in the male genitalia form was sufficient for diagnosis of both species. Females of *M. barclayi* sp. n. cannot be identified unless associated with males from the same collecting event.

### Mesomerodon gilletti

Taxon classificationAnimaliaORDOFAMILIA

Soula, 2008

Figs 4–5 , 8 , 9–13 , 16–17 , 18 , 23 , 26

Mesomerodon gilletti Soula, 2008: 21 (original combination)

#### Type material.

Holotype male, allotype female, and eight paratypes (four male, four females. Holotype male at CCECL with label data: a) “Tena (E), 9/91, (750m)” (handwritten), b) male genitalia card mounted c) “Holotype, 2007, Mesomerodon gilletti S., Soula” (typeset and handwritten on red label). Allotype female at CCECL with label data: a) “Tena (E), 9/91, (750m)” (handwritten), b) “Allotype, 2007, Mesomerodon gilletti S., Soula” (typeset and handwritten on red label). Paratype male at CCECL with label data: a) “Tena (E), 9/91, (750m)” (handwritten), b) male genitalia card mounted, c) “Paratype, 2007, Mesomerodon gilletti S., Soula” (typeset and handwritten on red label). Paratypes (2 females) at CCECL with label data: a) “Tena (E), 9/91, (750m)” (handwritten), b) “Paratype, 2007, Mesomerodon gilletti S., Soula” (typeset and handwritten on red label). Paratypes (2 males) at CCECL with label data: a) “Misahuali (E.), 5/91” or “Misahuali (Eq.), 5/91” (handwritten), b) male genitalia card mounted, c) “Paratype, 2007, Mesomerodon gilletti S., Soula” (typeset and handwritten on red label). Paratype male at CCECL with label data: a) “EQUATEUR: Prov. NAPO, MISAHUALLI ile ANACONDA, Alt. 350 m.; 17–22.9.1990, Leg. Joss” (typeset), b) male genitalia card mounted, c) “Paratype, 2007, Mesomerodon gilletti S., Soula” (typeset and handwritten on red label). Paratypes (2 females) at CCECL with label data: a) “Misahuali (E.), 5/91” (handwritten), b) “Paratype, 2007, Mesomerodon gilletti S., Soula” (typeset and handwritten on red label).

#### Description

**(based on 50 males and 19 females).** Length from apex of clypeus to apex of pygidium 18.0–20.0 mm (♂) and 21.0–24.0 mm (♀); width at mid-elytra 10.0–11.0 mm (♂) and 12.0–14.0 mm (♀). Legs: Protarsomere 2 of male lacking well-defined striae at ventral apex (Fig. 26). Male genitalia (Figs 23): Parameres with elongate, narrow projection (=stem) and with nearly obsolete impressed longitudinal fissure (Fig. 23a); stem broad, not narrowed toward apex, subapex lacking paired, spinose projections; ventral sclerite of phallobase with surface concave, apex quadrate (Fig. 23c); lateral view diagnostic (Fig. 23b).

#### Diagnosis.

*Mesomerodon gilletti* males are differentiated from other *Mesomerodon* species by the following combination of characters: Protarsomere 2 of male ventrally with striated region poorly defined or lacking at apex (striated in *M. spinipenne*; lacking in *M. barclayi* sp. n.; [Figs 25–27]) and parameres, and ventral sclerite of phallobase (Fig. 23 versus Fig. 24 in *M. spinipenne* and Fig. 22 in *M. barclayi* sp. n.). *Mesomerodon gilletti* females can only be confidently determined when associated with male specimens from the same collecting event.

#### Distribution

(Fig. 18). Known from western (lowland) Amazonia in Ecuador and Colombia (new country record). The species occurs in apparent sympatry with *M. barclayi* sp. n. A record of ‘*Mesomerodon* species 1’ from Napo Province in Paucar-Cabrera (2005) probably represents a record of *Mesomerodon gilletti*. Records of *Mesomerodon* from Ecuador (Blackwelder 1944, Ohaus 1918, 1934, 1952, Machatschke 1972, Moore et al. 2017, Paucar-Cabrera 2005) are records for either *M. gilletti* or *M. barclayi* sp. n.

#### Locality data

**(Suppl. material 1).** 69 specimens from 12 collections.

COLOMBIA: Putumayo

ECUADOR: Napo, Orellana, Sucumbíos

#### Temporal data.

Based on label data, this species is known to be active in the months of February, May, June, August, September, October, and November.

#### Natural history.

Based on label data, adult *M. gilletti* are found associated with lights at night. Immature stages are unknown.

#### Remarks.

Soula (2008) named this species based on twelve specimens, dedicating the species to Conrad Gillett (then at the BMNH) who provided specimens for Soula’s study. In overall appearance, *M. gilletti* was not discernable from *M. spinipenne* except for the “parameres that are different enough to justify the status of the species in its own right” (Soula 2008).

### Mesomerodon spinipenne

Taxon classificationAnimaliaORDOFAMILIA

Ohaus, 1905

Figs 6 , 7 , 14 , 18 , 24 , 27

Mesomerodon spinipenne Ohaus, 1905: 320–321 (original combination).

#### Type material.

Lectotype male (designated by Soula 2008) and three paralectotypes (1 male, 2 females). Lectotype male at ZMHB with label data: a) “bei Pozuzu, Eckardt S.” and “O. Peru, Chuchurras” (handwritten on verse and obverse), b) “Mesomerodon spinipenne, Type ♂ Ohs.” (handwritten on red label), c) “SYNTYPUS, Mesomerodon spinipenne Ohaus, 1905, labelled by MNHUB 2007” (typeset on red label), d) male genitalia card mounted, e) “Lectotype Mesomerodon spinipenne Oh., 2007 Soula” (typeset and handwritten on red label). Paralectotype female at ZMHB with label data: a) “60.” (handwritten, one egg mounted), b) “BRAZIL R.Purus” (typeset), c) “Mesomerodon spinipenne ♀, Cotype Ohs.” (handwritten on red label), d) “SYNTYPUS, Mesomerodon spinipenne Ohaus, 1905, labelled by MNHUB 2007” (typeset), e) “Paralectotype 2007, Mesomerodon spinipenne Oh. Soula det.” (typeset and handwritten on red label). Paralectotype female at ZMHB with label data: a) “Chuchuras, Amazonas” (handwritten), b) “Mesomerodon spinipenne, Cotype ♀ Ohs.” (handwritten on red label), c) “SYNTYPUS, Mesomerodon spinipenne Ohaus, 1905, labelled by MNHUB 2007” (typeset on red label), d) “Paralectotype 2007, Mesomerodon spinipenne S., Soula det.” (typeset and handwritten on red label). Paralectotype male deposited at BMNH with label data: a) “Chuchurras Peru” (handwritten), b) “Ohaus determ., Mesomerodon spinipenne Ohs.” (typeset and handwritten), c) “Peru 1907•27” (handwritten), d) “Co-type” (typeset on round yellow label), d) “♂” (typeset), e) “Cotypus!” (typeset on red label), f) male genitalia card mounted, g) “Paralectotype, Mesomerodon spinipenne, Ohaus, 1905, M Seidel des. 2016” (typeset on red label).

Ohaus (1905: 321) stated that the type series included at least one male and at least one female (but likely at least two females based on the length/width range that Ohaus provided) from “Peru Chuchuras (Eckhard); Amazonas, Rio Purus.” We found one additional male syntype at the BMNH that fits the measurements provided by Ohaus (1905), and we labeled it as a paralectotype. It is possible that additional paralectotypes may be found in collections.

#### Description

**(based on 52 males and 161 females).** Length from apex of clypeus to apex of pygidium 17.0–20.0 mm (♂) and 19.0–24.0 mm (♀); width at mid-elytra 10.0–12.0 mm (♂) and 11.0–14.0 mm (♀). Legs: Protarsomere 2 (♂) with well-defined striae at ventral apex (Fig. 27). Male genitalia (Fig. 24): Parameres with elongate, narrow projection (=stem) and with longitudinal, impressed fissure (Fig. 24a); stem broadened abruptly at apex, subapex with paired, spinose projections (almost appearing broken); ventral sclerite of phallobase with surface concave, apex quadrate (Fig. 24c); lateral view diagnostic (Fig. 24b).

#### Diagnosis.

*Mesomerodon spinipenne* males are differentiated from other *Mesomerodon* species by the following combination of characters: Protarsomere 2 with well-defined striae at ventral apex (Fig. 27) (lacking striae in *M. gilletti* and *M. barclayi* sp. n.; Figs 25–26) and form of the parameres, and form of the ventral sclerite of the phallobase (Fig. 24 versus Fig. 23 in *M. gilletti* and Fig. 22 in *M. barclayi* sp. n.). Females can only be confidently determined when associated with male specimens from the same collecting event. Females occurring in Bolivia and Peru are most likely conspecific with *M. spinipenne* (Figs 6–7).

#### Distribution

(Fig. 18). *Mesomerodon spinipenne* is the most broadly distributed species in the genus, and it occurs from central Peru to central Bolivia in the western (lowland) Amazonia (from 230 to 762 m elevation). The species has been recorded from Brazil, “Rio Purus” (Blackwelder 1944, Ohaus 1905, 1934, 1952, Machatschke 1972, Krajcik 2008, Soula 2008, Moore et al. 2017), but in our view this is an erroneous record. The record is based on a paralectotype female collected at the Rio Purus that rises in the Uyacali Region in Peru and flows into Brazil and which could, therefore, have been collected either in Peru or Brazil. Based on our examination of 213 specimens, we think that this specimen represents a Peruvian locality. Records of the species from Ecuador (Blackwelder 1944, Ohaus 1918, 1934, 1952, Machatschke 1972, Moore et al. 2017, Paucar-Cabrera 2005) are incorrect. These are records for either *M. gilletti* or *M. barclayi* sp. n.

#### Locality data

**(Suppl. material 1).** 213 specimens from 18 collections.

BOLIVIA: Cochabamba (Chapare), Huánuco (Blackwelder 1944, Ohaus 1905, 1918, 1934, 1952, Machatschke 1972, Soula 2008, Ratcliffe et al. 2015), Junín (Blackwelder 1944, Ohaus 1905, 1918, 1934, 1952, Machatschke 1972, Soula 2008, Ratcliffe et al. 2015), Pasco (Blackwelder 1944, Ohaus 1905, 1918, 1934, 1952, Machatschke 1972, Soula 2008, Ratcliffe et al. 2015), Santa Cruz (Ichilo, Sara)

PERU: Ayacucho (La Mar), Cusco (Quispicanchi), Huánuco (Leoncio Prado, Puerto Inca), Madre De Dios (Manú, Tambopata), Pasco (Oxapampa), Ucayali (Padre Abad), Santa Cruz (Blackwelder 1944, Ohaus 1918, 1934, 1952, Gutiérrez 1951, Machatschke 1972, Moore et al. 2017).

#### Temporal data.

Based on label data, this species is known to be active in all months except January and February.

#### Natural history.

Based on label data, adult *M. spinipenne* are active at night and can be collected at lights. Immature stages are unknown.

#### Remarks.

Based on body measurements provided by Ohaus (1905), we conclude that he had a minimum of 3 specimens: 1 male (length 18.5mm, width 10.5mm) and 2 females (length 22–23.5mm, width 12.5–13.5mm) from two localities (Chuchuras and Rio Purus). The lectotype specimen at ZMHB was designated by Soula (2008), and it is a male specimen labeled by Ohaus as “type” (“Mesomerodon spinipenne, Type ♂ Ohs.”) and with the locality label “bei Pozuzu, Eckardt S.” and “O. Peru, Chuchurras”. As part of this research, we found and labeled specimens that were invalidly labeled as type specimens and that do not belong to the type series (see “Type Specimens”).

It is possible that the specific epithet, “*spinipenne*”, refers to the apex of the elytra in the male which possess an apical spine or tubercle. The Latin root “*spini*” refers to spine or thorn, and the Latin root “*penna*” refers to wing. This character state is not unique to *M. spinipenne*; instead, it is a synapomorphy for all species in the genus.

## Discussion

The genus *Mesomerodon* is composed of three very similar species, two of which that have evaded discovery since the description of the genus by Ohaus (1905). We discovered specimens of our new species, *M. barclayi* sp. n. in collections that were studied by Ohaus and Soula, both experts who were not able to detect this cryptic species based on external features. Stasis in external morphology, in combination with the apparent sympatry of two *Mesomerodon* species in Ecuador, corroborate two hypotheses for generation of cryptic species: nonvisual mating signals and ecological specialization in similar niches (Bickford et al. 2007). Sympatric distribution and external similarity of *M. barclayi* sp. n. and *M. gilletti* suggest that sexual selection might be a driver for diversification in the genus. Only aedeagal characters differ between species, thus sexual selection by female choice may drive the evolution of male genitalia (Eberhard 1985), and this could be accompanied with differences in mating pheromones or mating calls (Bickford et al. 2007). It is possible that specialization in food plants or other life history-dependent factors may drive diversification. Studies of presumed dietary generalists in narrow ecological regions have revealed cryptic beetle and butterfly species complexes with dietary specializations (Blair et al. 2005, Hebert et al. 2004).

Co-distributed cryptic species complexes may be a function of the western (lowland) Amazonian region with its aseasonal climate, humid forest, and heterogeneous vegetation. The distribution of two species of *Mesomerodon* in Ecuador coincides with highest global diversity of passerine birds and anurans (InfoNatura 2007) as well as the region for highest wood biomass productivity (Hoorn and Wesselingh 2010). The Ecuadorian Amazonian region descends from the foothills to elevations of 200–400 m and receives approximately 282 cm of precipitation annually (Dangles et al. 2009). The absence of a prolonged dry season, varied topography, and warm temperatures make the region a hotspot for biodiversity (Myers et al. 2000). In this region, small differences in elevation and vegetational cover create refuges (Dangles et al. 2009) that may allow for ecological niche diversification, especially for herbivorous species such as those in the genus *Mesomerodon*.

Future studies associated with these cryptic species are needed to examine divergence, population structure, and sister group relationships of the genus. Molecular data will allow association of males and females for each species. Focused fieldwork could determine distributional limits and yield ecological data which will assist in understanding the origin and cause of sympatry.

## Supplementary Material

XML Treatment for Mesomerodon

XML Treatment for Mesomerodon barclayi

XML Treatment for Mesomerodon gilletti

XML Treatment for Mesomerodon spinipenne

## Supplementary materials

Supplementary material 1 Distribution data for *Mesomerodon* species (Coleoptera, Scarabaeidae, Rutelinae) Data type: occurrence data Explanation note: Specimen level data used for niche model, phenology, and overall distribution. This dataset is made available under the Open Database License (http://opendatacommons.org/licenses/odbl/1.0/). The Open Database License (ODbL) is a license agreement intended to allow users to freely share, modify, and use this Dataset while maintaining this same freedom for others, provided that the original source and author(s) are credited.Matthias Seidel, Mary L. Jameson, Rachel L. Stone
